# Liver Metastasectomy to Obtain Tumor-Infiltrating Lymphocytes: Technical Considerations and Report on Single-Center Experience

**DOI:** 10.1245/s10434-026-19242-8

**Published:** 2026-02-20

**Authors:** Lisa M. Kenney, Aaron J. Dinerman, Alexandra M. Gustafson, Abraham A. Hakim, Stephanie L. Goff, Mei Li M. Kwong, Udai S. Kammula, Jonathan M. Hernandez, James C. Yang, Steven A. Rosenberg, Nicholas D. Klemen

**Affiliations:** 1 Surgery Branch, Center for Cancer Research, National Cancer Institute, National Institutes of Health, Bethesda, MD USA; 2 Department of Surgery, UPMC Hillman Cancer Center, University of Pittsburgh, Pittsburgh, PA USA; 3 Surgical Oncology Program, Center for Cancer Research, National Cancer Institute, National Institutes of Health, Bethesda, MD USA

**Keywords:** Liver metastasectomy, Tumor-infiltrating lymphocytes, Adoptive cell therapy, Metastatic cancer, Hepatobiliary surgery, Immunotherapy, Surgical oncology, Tumor-infiltrating lymphocytes procurement

## Abstract

**Background:**

Adoptive cell transfer (ACT) of tumor-infiltrating lymphocytes (TIL) is an FDA-approved treatment for metastatic melanoma and continues to be studied in other cancers. As a common site of metastatic disease, the liver is potentially a useful site for TIL procurement, but only if the procedures can be performed with an acceptably low risk of complications.

**Methods:**

A retrospective analysis was performed on 158 patients who underwent liver resection for TIL procurement between 2000 and 2024. Patients had metastatic melanoma or epithelial cancers. The primary outcomes were 30 days and 90 days morbidity and mortality.

**Results:**

Liver resection for TIL was performed in 81 patients with melanoma and 77 patients with epithelial cancers. The overall 30 days and 90 days mortality were 1% and 10%, respectively, and no deaths were attributed to surgery. Complication rates (Clavien-Dindo ≥ III) were 5% and 7%, respectively. Viable tumor was confirmed in 100% of resected lesions.

**Conclusions:**

Liver resection for TIL procurement is feasible and safe in carefully selected patients. Candidate patients should be discussed in a multidisciplinary setting with both medical and surgical expertise reaching a consensus on the optimal site selection to minimize risk and maximize therapeutic success.

Adoptive cell transfer (ACT) of tumor-infiltrating lymphocytes (TIL) can mediate durable complete tumor regression in patients with metastatic melanoma.^1^ In 2024, TIL was FDA-approved for the treatment of metastatic melanoma after a single arm phase II trial demonstrated it was effective in patients who were refractory to PD-1 blockade.^2,3^ Tumor-infiltrating lymphocytes is available via special hospital exemptions in some European countries based on a randomized phase III trial comparing locally manufactured TIL to ipilimumab.^4^ Tumor-infiltrating lymphocytes is also an investigational therapy for patients with epithelial cancers in ongoing clinical trials.^5–8^

Metastasectomy to procure a tumor for ex vivo TIL culture is a necessary first step to build this therapy. Patient selection and technical considerations for surgical TIL procurement has been previously described.^9,10^ A recent review by Mullinax and colleagues offers a helpful overview of surgical considerations reflecting the growing institutional experience with TIL therapy and represents a valuable step toward standardizing practices.^11^

Because the liver is a common site for metastatic disease, hepatic resection has potential to be a useful site for TIL procurement, but only if the procedures can be performed with an acceptably low risk of complications. The liver is also a complicated site for TIL procurement, because potentially useful metastases may be intermixed with benign or previously treated lesions, and prior surgery and/or instrumentation of the biliary tree has potential implications for tumor harvest.

A preliminary experience of hepatic metastasectomy for TIL procurement was previously reported.^12^ However, the small sample size limited its power to estimate the risk of rare adverse events. Moreover, during the interceding 12 years since its publication, institutional practices pertaining to selection of patients and lesions for harvest have been refined. This study was undertaken to extend the original report and systematically evaluate our experience with liver resection performed for the purpose of TIL procurement since 2004 to assess safety and to detail our approach to patient and lesion selection.

## Patient and Lesion Selection

The screening process for liver TIL harvest begins by evaluating whether patients are candidates for cell therapy. While protocols may vary, in general, the appropriate candidates have good performance status, normal basic laboratory values, and lack of major medical comorbidities, active infections, or uncontrolled brain metastases.

Once a patient is determined to be a candidate for cell therapy, the team identifies a feasible metastatic lesion for procurement. Nonvisceral TIL harvests are prioritized if they can be performed with minimal morbidity, such as excision of axillary, cervical, or inguinal lymph nodes, or skin/soft tissue metastases. If such sites are not feasible, peritoneal metastases that can be recovered via laparoscopic surgery or peripheral lung lesions amenable to video-assisted thoracoscopic surgery are considered. If there are no other suitable anatomical sites with resectable tumors, hepatic TIL procurement is considered.

Patients being considered for liver tumor procurement are evaluated comprehensively with a physical examination and cross-sectional imaging using a multiphase computed tomography (CT) of the abdomen and pelvis with arterial, portal venous, and delayed phases. Most patients also undergo liver-specific imaging with abdominal magnetic resonance imaging and positron emission tomography (PET) if needed. Additional diagnostic studies are tailored based on clinical context.

Due to the potential risks of liver procurement, it is key to select patients who are highly likely to remain eligible for cell therapy even several months after surgery. For this, particular attention is paid to the patient’s oncologic history. Liver procurement is avoided in individuals with an extensive intra-abdominal surgical history, and those who may experience rapid disease progression that might preclude later treatment. Liver harvests are mainly limited to patients who have at least one of the following characteristics—and ideally multiple: (1) a low burden of metastatic disease; (2) serial imaging demonstrating relatively indolent disease; and/or (3) potentially effective bridging systemic therapy.

Next, usual liver-specific factors are considered before planning TIL procurement. Underlying liver health, such as the absence of hepatic disease, portal hypertension, or sequelae of regional therapies (e.g., chemoembolization or liver-directed radiation) is confirmed. Imaging is evaluated for evidence of biliary ductal dilation or regional perfusion abnormalities.

Several lesion-specific factors are potentially important for procurement. While tumors should be at least 1.5 cm in size, there is no benefit from removing lesions that are substantially larger. Lesions ideally should not be adjacent to major vascular or biliary structures to minimize the risk of operative complications. It is also critical to ensure the lesion being procured is confirmed or highly likely to represent metastatic disease, and that the tumor itself is healthy and viable. This assessment can be challenging, because benign hepatic lesions can be intermixed among metastases and because previous systemic, regional, or local therapies can result in tumors that are largely nonviable due to treatment effect. For example, lesions previously treated with ablation, SBRT, or Y-90 may lack the necessary lymphocyte infiltration and be unsuitable for TIL generation. Similarly, tumors with ongoing responses to prior systemic therapy might not yield sufficient viable tumor tissue.

The usual strategy to confirm a putative harvest site represents a viable metastasis is to identify imaging characteristics of a metastatic lesion plus unequivocal demonstration of growth on serial imaging. In the absence of growth, imaging can be highly suggestive of a metastasis, but caution is warranted if the patient has received recent treatment for their cancer, because it could represent a nonviable metastasis with treatment effect. In these circumstances, it is reasonable to wait for an interval of 6–8 weeks to confirm growth or to perform a biopsy either preoperatively or intraoperatively to confirm viable cancer before committing to a full resection.

Patients selected for hepatic metastasectomy are advised to hold chemotherapy for 4 weeks and anti-VEGF therapy for 6 weeks prior to TIL harvest. Patients on anticoagulation are advised to hold these medications several days before surgery. It is also important to advise the managing anesthesiologist to avoid steroid administration in patients undergoing procurement as there is a theoretical chance the steroids could suppress growth of the lymphocytes during *ex vivo* culture. After surgery, patients are advised to resume chemotherapy as early as 10–14 days at the discretion of their oncologist, assuming a normal postoperative recovery.

## Methods

A retrospective review of a prospectively collected database was performed to identify all patients who underwent liver metastasectomy for melanoma or epithelial cancers in the context of a clinical trial protocol at the National Cancer Institute (NCI) from September 2004 until December 2024.

Patient information such as demographics, prior surgical history, active medications at the time of screening, stage of cancer at diagnosis, and number of prior chemotherapy regimens were gathered from medical records. Tumor and liver characteristics such as size of lesions and distribution of liver metastases were analyzed via CT, magnetic resonance imaging, and PET imaging (when available). Operative and pathology reports were reviewed for surgical details and confirmation of metastatic disease upon TIL harvest. Operative outcomes (including 30 day and 90 day mortality events) were obtained by review of inpatient and outpatient medical records, with Clavien-Dindo grades assigned retrospectively. Time to postoperative surgical clearance was reported rather than length of stay as patients often remained admitted for other trial-related activities.

All patients signed institutional review board-approved consents for tissue procurement. Eligible patients were 18 years of age or older (17 years of age when accompanied by a legal guardian), negative for HIV as well as hepatitis B and C, with a good performance status with an Eastern Cooperative Oncology Group (ECOG) status of ≤1.

### Statistical Analysis

Continuous data are reported as medians with 25th and 75th interquartile range (IQR) with comparisons between groups performed using Wilcoxon rank-sum tests and Kruskal-Wallis H tests. Categorical data are presented as proportions and tested for significance using Fisher’s exact test and chi-square tests where indicated, all as univariate analyses. All tests used a two-sided uncorrected *p* value < 0.05 as a level of statistical significance.

## Results

### Patient Characteristics

A total of 158 patients, including 81 with metastatic melanoma and 77 with epithelial cancers, underwent hepatic resection for TIL harvest (Table 1). The median age at time of surgery was 50 years [interquartile range (IQR), 40–56 years] and 52 [IQR, 42–58 years] for the melanoma and epithelial cohorts respectively. A total of 143 patients (91%) self-identified as Caucasian race, nine patients (6%) identified as Asian race, two patients (1%) identified as Black race, two patients (1%) identified as “multiple” or “other” race, and two patients’ (1%) race was not recorded. Most patients in both the melanoma (88%) and epithelial cancer cohorts (82%) had an ECOG status of 0. Liver-only metastatic disease was present in 33% of patients with melanoma and 18% of those with epithelial cancer, with extensive bilobar distribution of tumor burden common in both groups. The specific histologies in the epithelial cancer cohort varied, with breast cancer in 26 (34%) patients, colorectal cancer (CRC) in 23 (30%) patients, cholangiocarcinoma in nine (12%) patients, pancreatic ductal adenocarcinoma (PDAC) in eight (10%) patients, and other epithelial cancers in 11 (14%) patients.

**Table 1:** Clinical Characteristics of 158 patients with metastatic melanoma and epithelial cancer who underwent liver resection for ACT therapy

Characteristic	Melanoma cohort	Epithelial cohort
Number of patients	81	77
Age, median (range) at operation (years)	50 (17–67)	51 (29–67)
*Sex*		
Male	51 (63%)	28 (36.4%)
Female	30 (37%)	49 (63.6%)
ECOG status 0	71 (87.7%)	60 (81.8%)
*Melanoma subtypes*		
Cutaneous melanoma	59 (72.8%)	0
Ocular melanoma	21 (25.9%)	0
Vulvar melanoma	1 (1.2%)	0
Breast Cancer	0	26 (33.8%)
Colorectal Cancer	0	23 (29.9%)
Colon Cancer	0	14 (18.2%)
Rectal Cancer	0	9 (11.7%)
Cholangiocarcinoma	0	9 (11.7%)
Pancreas Cancer	0	8 (10.4%)
Other epithelial cancers*	0	11 (14.3%)
*Stage at Diagnosis*		
Stage 1 or 2	36 (44.4%)	14 (18.2%)
Stage 3	18 (22.2%)	13 (16.9%)
Stage 4	14 (17.3%)	46 (59.7%)
N/A (not stage 4)	13 (16.4%)	4 (5.2%)
Liver-only metastatic disease	27 (33.3%)	14 (18.2%)
Pre-operative carcinomatosis	3 (3.7%)	4 (5.2%)
Pre-operative ascites	2 (2.5%)	2 (2.6%)
Prior systemic treatment	52 (64.2%)	76 (98.7%)
Number of systemic regimens, median (IQR)	1 (0 - 2)	3 (2 - 4)
Chemotherapy	8 (9.9%)	75 (97.4%)
Checkpoint inhibitor	18 (22.2%)	11 (14.3%)
IL-2	18 (22.2%)	0
Days from last systemic therapy to surgery, median (IQR)	72 (50 - 193)	59 (39.5 - 89.5)
Self-reported recent weight loss (previous 3 months)	6 (7.4%)	9 (11.7%)
Anticoagulation	2 (2.5%)	10 (13%)

In the melanoma cohort, patients had a median of one and maximum of four prior systemic regimens (IQR 1–3) (Table 1). Of note, only 18 (22%) patients with melanoma had prior immune checkpoint blockade (anti-PD-1, CTLA-4 or PD-L1). In the epithelial cohort, patients were more heavily pretreated with a median of three prior systemic regimens (IQR, 2–4), including 44% who had prior exposure to oxaliplatin or irinotecan-based chemotherapy. Eleven (14%) patients in the epithelial cohort were previously treated with checkpoint blockade. The median interval from last systemic therapy to surgery was 72 days for patients with melanoma and 59 days for those with epithelial cancer.

### Liver Characteristics

Many patients in both groups had extensive intrahepatic disease, exceeding ten individual liver metastases in 42% of melanoma and 77% of epithelial cancer cases (Table 2). A large tumor burden—approximately defined as having the sum of the five largest lesions exceeding 10 cm—was also common.

**Table 2:** Liver Characteristics of 158 patients with metastatic melanoma and epithelial cancer who underwent liver resection for ACT therapy

Characteristic	Melanoma cohort (81 patients)	Epithelial cohort (77 patients)
*Number of Liver metastases*		
1	10 (12.3%)	9 (11.7%)
2-5	30 (37%)	22 (28.6%)
6-9	7 (8.6%)	12 (15.6%)
≥10	34 (42%)	34 (77.3%)
Liver tumor burden (sum of largest 5 lesions)		
<5 cm	15 (18.5%)	17 (22.1%)
5–10 cm	23 (28.4%)	27 (35.1%)
>10 cm	43 (53.1%)	33 (42.9%)
*Distribution of liver metastases*		
Right side only	12 (14.8%)	15 (19.5%)
Left side only	6 (7.4%)	5 (6.5%)
Bilobar	63 (77.8%)	57 (74%)
Prior liver resection	2 (2.5%)	7 (9.1%)
Prior biliary stent in situ	0	2 (2.6%)
Pre-op biliary dilation	1 (1.2%)	7 (9.1%)
Pre-op splenic vein thrombosis	0	1 (1.3%)
Prior liver-directed radiation therapy	3 (3.7%)	4 (5.2%)
Prior liver-directed ablation therapy	3 (3.7%)	5 (6.5%)
Exposure to oxaliplatin or irinotecan therapy	0	34 (44.2%)

A small number of patients had undergone previous liver resection (2 melanoma, 7 epithelial) or had been treated with prior liver-directed therapies, such as Y-90 radiation and ablation (6 melanoma, 9 epithelial), but these previously treated lesions were not used for TIL harvest (Table 2). No patients with melanoma had preoperative biliary stents, whereas two patients (3%) in the epithelial cohort had a biliary stent *in situ* and as detailed below, both developed abscesses after surgery. One patient with melanoma and seven with epithelial carcinoma had evidence of biliary dilation. These results together show that while many patients had a substantial burden of hepatic disease, most had no prior history of biliary instrumentation or liver-directed therapy.

**Fig. 1:**
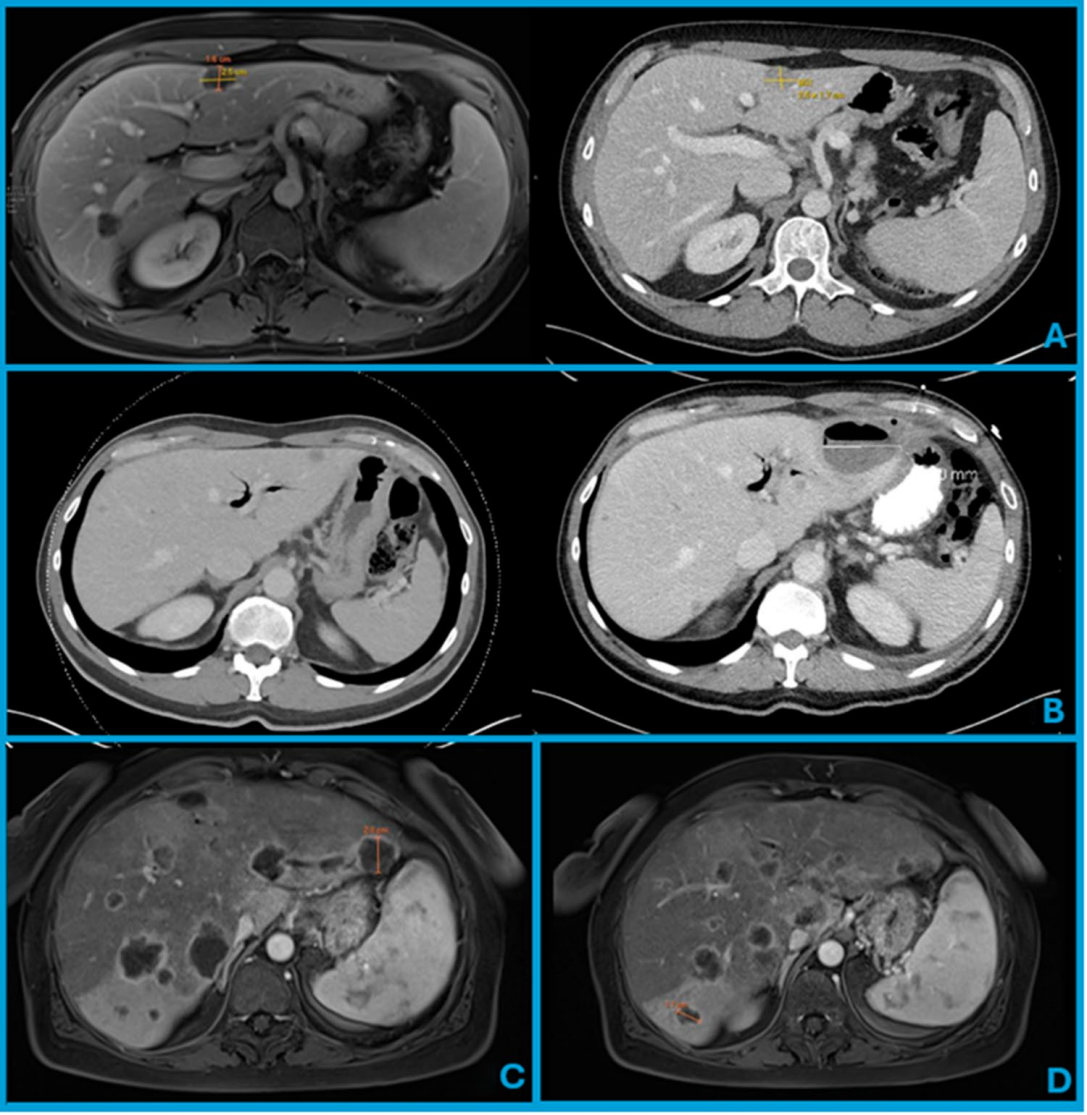
**A** Sequential imaging studies two months apart showing a non-progressing metastatic liver lesion secondary to treatment response that was found by laparoscopic biopsy to be necrotic and nonviable. The planned liver resection was aborted. **B** CT scan showing left-sided pneumobilia due to the presence of a biliary stent and a segment II liver lesion that was resected for TIL (left). The patient subsequently developed an abscess at the resection site requiring placement of a percutaneous drain (right). **C** Magnetic resonance imaging showing a metastatic liver lesion in segment II/III with a dilated proximal bile duct. **D** Magnetic resonance imaging showing a metastatic liver lesion in segment VI with a regional perfusion defect due to vascular involvement of proximal metastases

In 90% of patients, the site of procurement was predicted to have viable cancer by confirming imaging was consistent with a metastasis plus documented lesion growth on serial imaging studies. This approach avoided resection of stable or regressing lesions that may not have yielded viable tumor tissue. In a smaller subset of equivocal cases (11% of melanoma and 9% of epithelial), PET imaging was used to support lesion selection. In very rare instances, a biopsy of the lesion in question was necessary. In one anecdotal case, an intraoperative biopsy was performed of a known metastasis that was stable after systemic chemotherapy, which showed no viable tumor, and so the planned hepatic resection was aborted (Fig. 1A). Using this multimodal imaging-based approach, 100% of all resected hepatic specimens were confirmed to have viable metastatic cancer on final pathology.

### Surgical Technique

In the melanoma cohort, the most common surgical approach was open laparotomy (63%). All minimally invasive melanoma resections (*n* = 28) were performed by using a laparoscopic approach, as these occurred in an era (beginning in September 2004) before robotic assistance was available (Table 3). Minimally invasive surgery was used in 55% of patients in the epithelial cancer cohort as these occurred later (IQR 7/2015–9/2022), enabling a robotic-assisted approach in 20 of 42 minimally invasive cases.

**Table 3:** Surgical Technique for 158 patients with metastatic melanoma and epithelial cancer who underwent liver resection for ACT therapy

Characteristic	Melanoma cohort (81 patients)	Epithelial cohort (77 patients)
Date range of operations (IQR)	4/2008–8/2013	7/2015–9/2022
*Surgical Approach*		
Open/Laparotomy	51 (63%)	31 (40.3%)
Minimally Invasive	28 (34.6%)	42 (54.5%)
Laparoscopic	28 (34.6%)	21 (27.3%)
Robotic-assisted	0	20 (26%)
Hand-assisted	0	1 (1.3%)
Laparoscopic converted to Open	2 (2.5%)	4 (5.2%)
*Wedge vs anatomic resection*		
Wedge	54 (66.7%)	63 (81.8%)
Anatomic	25 (30.9%)	12 (15.6%)
Combination	2 (2.5%)	2 (2.6%)
Duration of surgery, median (IQR), minutes	172.5 (128.75 - 216.25)	139 (114–197)
*Use of pringle maneuver*		
Yes	37 (45.7%)	18 (23.4%)
total minutes of use, median (IQR)	10 (8–14.75)	15 (10–20)
No	44 (54.3%)	59 (76.6%)
*Number of wedges/specimens*		
1	70 (86.4%)	59 (76.6%)
2	9 (11.1%)	13 (16.9%)
3 or more	2 (2.5%)	5 (6.5%)
*Additional procedure performed at the time of liver resection*		
Cholecystectomy	18 (22.2%)	16 (20.8%)
Omental resection/peritoneal biopsy	9 (11.1%)	3 (3.9%)
Lymphadenectomy	3 (3.7%)	4 (5.2%)
Splenectomy	1 (1.2%)	9 (11.7%)
Colon resection	2 (2.5%)	0
LOA	1 (1.2%)	0
Other	1 (1.2%)	2 (2.6%)

**Fig. 2:**
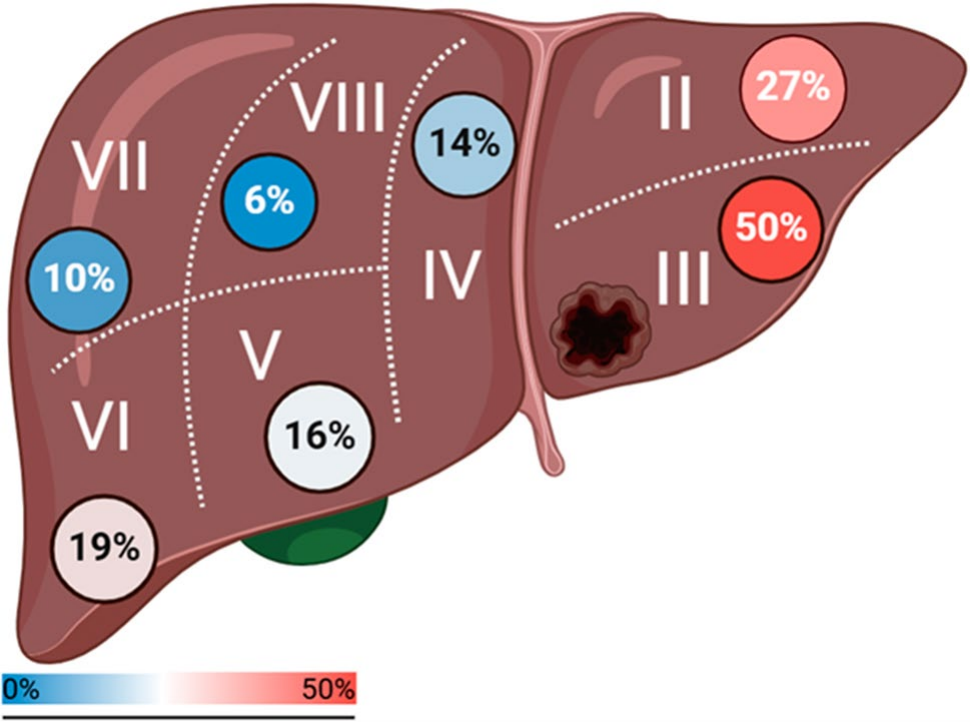
Heat map of a liver demonstrating involvement of each segment in liver TIL harvest for ACT in 158 patients with metastatic melanoma and epithelial cancers

Most resections in both cohorts were nonanatomic wedge resections (67% in melanoma, 82% in epithelial cancer). Liver segment III was the most frequent anatomical segment (53% melanoma, 47% epithelial) (Fig. 2). The second most involved liver segment was segment II, followed by segment VI, V, IVA/B, then segments VII, and VIII. No TIL harvests were done by resection of caudate lesions.

The median operative time was longer in the melanoma cohort than the epithelial cohort (172.5 min vs 139 min), potentially due to more open procedures and anatomic resections (Table 3). A Pringle maneuver was required in 45% and 23% of cases, respectively, with median Pringle times being 10 min (IQR 8–14.75 min) for the melanoma cohort and 15 min (IQR 10–20 min) for epithelial cases. These short operative and Pringle times illustrate that most procured specimens were peripheral liver tumors without proximity to major vascular structures. Additional concurrently performed procedures were uncommon, though cholecystectomy or portal/hepatic lymph node biopsy was performed in a subset of patients.

### Operative Outcomes

The median time to surgical clearance for discharge was shorter in the epithelial cancer cohort (3 days, IQR 3–5) compared with the melanoma cohort (5 days, IQR 4–6 days), potentially due to the higher rate of minimally invasive procedures in patients with epithelial cancers (Table 4). Median estimated blood loss was 100 mL in the epithelial cohort (IQR 30–200 mL) versus 150 mL in the melanoma group (IQR 50–325 mL). A blood transfusion was required in seven (8.6%) of patients with melanoma and three (3.9%) of patients with epithelial cancer.

**Table 4:** Surgical Outcomes for 158 patients with metastatic melanoma and epithelial cancer who underwent liver resection for ACT therapy

Characteristic	Melanoma cohort (81 patients)	Epithelial cohort (77 patients)
Median time to medical clearance (IQR), days	5 (4–6)	3 (3–5)
EBL, median (IQR)	150 (50 mL–325 mL)	100 mL (30 mL–200 mL)
Intra-operative transfusions	7 (8.6%)	3 (3.9%)
Post-operative transfusions	7 (8.6%)	4 (5.2%)
Patients with post-operative complications	4 (4.9%)	5 (6.5%)
Clavien-Dindo Grade IIIa	0	1 (1.3%)
Clavien-Dindo Grade IIIb	0	4 (5.2%)
Biloma requiring drain placement	3 (3.7%)	4 (5.2%)
Arrhythmia requiring cardioversion	1 (1.2%)	0
Clavien-Dindo Grade IV	0	0
Clavien-Dindo Grade V	0	0
Procedure-related 30-day mortality	0	0
Overall 30 day mortality	1 (1.2%)	1 (1.3%)
Overall 90 day mortality	9 (11.1%)	7 (9.1%)

In the melanoma cohort, four (4.9%) patients experienced Clavien-Dindo Grade IIIb complications: three of which were bilomas requiring drainage under anesthesia, and one (1.2%) of which was a postoperative arrhythmia requiring cardioversion (Table 4). The 30 day mortality rate was 1.2% (*n* = 1), a case of respiratory failure related to IL-2 administration. The 90 day mortality rate was 11.1% (*n* = 9), all due to disease progression.

In the epithelial cancer cohort, postoperative complications included one (1.3%) Grade IIIa event (thoracentesis) and four (5.2%) Grade IIIb complications, all postoperative bilomas managed with drainage (Table 4). Both patients who had biliary stents *in situ* developed postoperative fluid collections requiring drainage (Fig. 1B). A single 30 day mortality event was attributed to liver failure from rapidly progressing disease, but this patient also had developed a postoperative bile leak.

The 90 day mortality was 9.1% (*n* = 7), all due to disease progression. An exploratory analysis showed patients who died within 90 days of surgery had more metastases (*p* = 0.008**) and a high tumor burden as defined earlier (*p* = 0.0021**) (Table 5). Of seven total patients who developed postoperative bile leak requiring drainage, one died within 90 days due to rapid progression of their metastatic disease. The other instances of postoperative bile leakage did not delay TIL infusion.

**Table 5:** Exploratory analysis of patients with 90-day mortality Melanoma and Epithelial Cohorts, 158 patients

Characteristic	# 90-day mortality events	Relative Risk (95% CI)	Significance (*p*)
*Number of liver metastases*			
≥10 lesions *n* = 68	12	1.16 (1.04–1.34)	0.008**
< 10 lesions *n* = 90	4		
*Liver tumor burden (sum of largest 5 lesions)*			
<5 cm *n* = 32	2		0.0021**
5-10 cm *n* = 75	0		
> 10 cm *n* = 51	14		
*Post-operative bile leak requring drain placement*			
yes *n* = 7	1	1.47 (0.24–8.18)	0.5336
no *n* = 151	15		
*Exposure to oxaliplatin or irinotecan therapy*			
yes *n* = 34	4	1.02 (0.92–1.24)	0.7502
no *n* = 124	12		
*Method of liver resection*			
wedge resection *n* = 117	11		0.1008
anatomic resection *n* = 37	4		
combination *n* = 1	1		
*Pre-operative weight loss*			
yes *n* = 15	2	1.37 (0.35–4.54)	0.6511
no *n* = 143	14		
*Pre-operative carcinomatosis*			
yes *n*= 7	1	1.48 (0.24–8.18)	0.5336
no *n* = 151	15		
*Pre-operative ascites*			
yes *n* = 4	0	0 (0–7.59)	>0.99
no *n* = 154	16		
*Pre-operative anticoagulation therapy*			
yes *n* = 12	2	1.78 (0.45–6.13)	0.3484
no *n* = 146	14		
*Blood transfusion (intra-operative or post-operative)*			
yes *n* = 17	4	2.73 (0.99–6.63)	0.0742
no *n* = 141	12		

### Subsequent Treatment with Adoptive Cell Transfer of TIL

The indication for the described hepatic resections was to generate experimental autologous cell products for patients with metastatic cancer. The extended time frame and range of cancer types did not allow in-depth analyses of cell therapy outcomes from within these cohorts, as the barriers to protocol entry evolved over time. Across the combined melanoma and epithelial cohorts (*n* = 158), patients could broadly be grouped into one of three categories: treated (*n* = 76), patient-related barrier to treatment (*n* = 40), or cell-related barrier to treatment (*n* = 42). Patient-related barriers most often reflected disease progression that rendered patients ineligible for adoptive cell transfer, while cell-related barriers included insufficient TIL growth or lack of reactivity during neoantigen testing. Clinical outcomes for the treated patients, including tumor responses, have been previously reported.^1,5,13^

Most patients with a cell-related barrier to entry were in the epithelial cohort (28/42, 67%), but this higher rate reflects the increased complexity of the epithelial workflow. By design, TIL were required to recognize mutated proteins (neoantigens) derived from epithelial tumors, and in several cases, TIL expanded successfully but were not infused owing to insufficient neoantigen-specific reactivity.^5,14^ Additionally, the added time required for neoantigen screening and the heterogeneity of epithelial cancer types, each with distinct disease kinetics and treatment histories, potentially contributed to a greater frequency of disease progression or other patient-related barriers to treatment. Overall, our findings across melanoma and epithelial cancers demonstrate that, in most cases, TIL can be reliably cultured ex vivo from hepatic metastases.

## Discussion

Given the recent FDA-approval of TIL for patients with treatment-refractory metastatic melanoma and multiple ongoing clinical studies of TIL therapy for epithelial cancers, there is a growing clinical need for surgical tumor procurement. Patients with metastatic disease have potentially significant competing risks and may have diminished physiologic reserve. These factors, in addition to the elevated risk associated with liver surgery necessitate meticulous clinical evaluation, careful patient selection, and unique technical considerations before surgery is attempted. Recent data has shown that hepatic metastases of epithelial cancers are less likely to yield robust TIL growth and/or neoantigen-specific reactivity compared with other anatomic sites of harvest, such as lungs or lymph nodes, although earlier data suggested similar patterns of growth and activity of TIL from melanoma liver metastases.^15,16^ Given the balance of these factors, special attention should be reserved when considering procurement of hepatic metastases, but our experience suggests it can be done safely in carefully selected patients.

While the morbidity of liver procurement was low, roughly 5% of patients experienced Clavien-Dindo grade III or higher complications. This can be potentially life threatening to patients with metastatic disease because it can result in a delay in the resumption of systemic therapy and also can compromise later eligibility for TIL therapy. Given these risks, it is crucial to select patients who can tolerate such a complication and fully recover, while remaining potentially eligible for TIL therapy down the line.

Even in patients with uncomplicated operations, TIL can require 1–3 months to manufacture, and tolerating adoptive cell transfer requires robust physiologic reserve. This is why we strive to select patients who are highly likely to retain an excellent performance status and eligibility for cell therapy at least 3 months after surgery. In this regard, 90-day mortality is a useful metric since patients who succumbed to their disease before that time were, in retrospect, failures of patient selection on our part. That 90-day mortality was associated with the number and burden of liver metastases provides an additional note of caution for patient selection.

Biliary ductal dilation (Fig. 1C) or proximal perfusion defects (Fig. 1D) are key considerations for hepatic TIL harvest. Leakage of bile is always a potential risk of liver surgery, but surgery for TIL procurement is unique because peripheral tumors are sometimes removed while proximal metastases remain in situ, which is virtually never the case in curative-intent operations. These proximal tumors have the potential to cause elevated biliary pressures and increase the risk of bile leakage. For this reason, we consider biliary ductal dilation proximal to the target lesion to be an absolute contraindication to procurement, and regional biliary dilation is a relative contraindication.

Finally, given our experience with two patients with biliary stents in situ, we consider the presence of a biliary stent and/or pneumobilia to be an absolute contraindication to TIL procurement. Relative contraindications are any history of biliary stenting, instrumentation or surgery, as these may allow for ascending infection into expected postoperative seromas.

## Conclusions

In highly selected patients, the liver can represent a safe and feasible site of tumor procurement. Overall, we report a low rate of complications and a high success rate of tumor procurement in a population with major competing risks and important liver-specific considerations reflective of their metastatic cancer. These results reflect evolving practices for patient selection over two decades that may limit generalizability. This single-center study is additionally limited by selection bias due to strict eligibility criteria. In addition, the retrospective nature of this study inherently limits causal inference and the ability to directly compare safety outcomes with other surgical or institutional approaches. In summary, the critical components of hepatic TIL procurement are meticulous patient selection, absence of non-hepatic alternative metastases, low-risk hepatic and lesion-specific factors, and unequivocal evidence of viable tissue in the target lesion. Formalized consensus guidelines for patient and lesion selection for TIL procurement could further optimize safety and efficacy.
